# Microcirculatory disturbance in acute liver injury

**DOI:** 10.3892/etm.2021.10028

**Published:** 2021-04-09

**Authors:** Akifumi Kuwano, Miho Kurokawa, Motoyuki Kohjima, Koji Imoto, Shigeki Tashiro, Hideo Suzuki, Masatake Tanaka, Seiji Okada, Masaki Kato, Yoshihiro Ogawa

**Affiliations:** 1 Department of Medicine and Bioregulatory Science, Graduate School of Medical Sciences, Kyushu University, Higashi-ku, Fukuoka 812-8582, Japan; 2 Department of Pathophysiology, Medical Institute of Bioregulation, Kyushu University, Higashi-ku, Fukuoka 812-8582, Japan; 3 Core Research for Evolutionary Science and Technology (CREST), Japan Agency for Medical Research and Development, Chiyoda-ku, Tokyo 100-0004, Japan

**Keywords:** acute liver failure, serum aminotransferase/lactate dehydrogenase ratio, sinusoidal hypercoagulation, intrahepatic hypoxia, microcirculatory disturbance

## Abstract

Microcirculatory disturbance is thought to be involved in the pathogenesis of acute liver injury (ALI). The current study examined the pathophysiologic role of hepatic microcirculatory disturbance in patients with ALI and in mouse models of ALI. Using serum aminotransferase (ALT)/lactate dehydrogenase (LDH) ratio as a hypoxic marker, 279 patients with ALI were classified into the low ALT/LDH ratio (ALT/LDH ≤1.5) and high ALT/LDH ratio group (ALT/LDH >1.5). In the low ALT/LDH ratio group, serum ALT, LDH, fibrinogen degradation products and prothrombin time-international normalized ratio were increased relative to the high ALT/LDH ratio group. Histologically, hepatic expression of tissue factor (TF) and hypoxia-related proteins was enhanced in the low ALT/LDH ratio group, and this was accompanied by sinusoidal fibrin deposition. Sinusoidal hypercoagulation and intrahepatic hypoxia was also analyzed in two different mouse models of ALI; Concanavalin A (ConA) mice and Galactosamine/tumor necrosis factor (TNF)-α (G/T) mice. Serum ALT/LDH ratio in ConA mice was significantly lower compared with G/T mice. Pimonidazole staining revealed the upregulation of hypoxia-related proteins in ConA mice. Recombinant human soluble thrombomodulin improved liver damage in ConA mice in association with reduced sinusoidal hypercoagulation and intrahepatic hypoxia. The present study provides evidence that serum ALT/LDH ratio aids in the identification of patients with ALI and intrahepatic hypoxia as a result of microcirculatory disturbance. The results facilitate the improved understanding of the pathogenesis of ALI, thereby offering a novel therapeutic strategy against ALI, which arises from sinusoidal hypercoagulation.

## Introduction

Acute livfer failure (ALF) is a life-threatening systemic disorder characterized by severe coagulopathy and encephalopathy (1). Currently, liver transplantation is the only therapeutic method proven to improve the prognosis of ALF patients. However, the pathogeneses of ALF is poorly understood (2,3). Rake *et al* previously reported sinusoidal hypercoagulation in the liver of ALF and demonstrated the usefulness of anticoagulation therapy in ALF patients (4). Thus, hepatic microcirculatory disturbance as well as excessive activation of immune cells have attracted interest as the pathogenesis of ALF (5). Massive hepatic necrosis and scarce regeneration, which occur in acute liver injury (ALI), result in acute depression of hepatic function or ALF. The histological findings of ALF and its clinical features including the sudden onset and aggressive expansion of liver damage suggest the involvement of blood perfusion disorders. Indeed, intravital microscopy analysis revealed a significant correlation between the extent of sinusoidal flow disturbance and hepatic tissue damage in animal studies of ALF (6-9), and sinusoidal fibrin deposition as a result of impaired coagulation system suggests the involvement of sinusoidal hypercoagulation to induce hepatic blood flow disturbance (10,11). However, the association between sinusoidal hypercoagulation and intrahepatic hypoxia in ALF has not been addressed, because there are no clinically applicable methods to assess sinusoidal perfusion. On the other hand, there is no significant correlation between the extent of liver damage and systemic coagulation disorder, and heparin treatment is not effective in paracetamol-induced ALF (10,12). It is, therefore, unclear whether hepatic microcirculatory disturbance and intrahepatic hypoxia occur in the liver of ALF and if so, how they affect the clinical features of ALF is poorly understood.

Lactate dehydrogenase (LDH) is a critical enzyme which catalyzes the conversion of pyruvate to lactate under hypoxic condition. Because LDH is transcriptionally upregulated when blood supply is insufficient, it is likely that LDH serves as a marker for tissue hypoxia (13-15). Interestingly, immunostaining with liver biopsy samples showed marked increase in LDH in ALF and cirrhosis but only slight increase in chronic hepatitis, suggesting intrahepatic hypoxia in the ALF liver (11). On the other hand, alanine aminotransferase (ALT) is known to be released from damaged hepatocytes, thereby serving as a maker of liver injury. We, therefore, hypothesize that serum ALT/LDH ratio reflects the hypoxia-induced liver damage, which can be clinically used for the evaluation of intrahepatic hypoxia. Indeed, several studies have reported serum ALT/LDH ratio reflects a hypoxic marker in the prognosis of ALI patients (16,17), but little has been reported on intrahepatic hypoxia classified serum ALT/LDH ratio histologically.

In this study, we classified ALI patients based upon serum ALT/LDH ratio and found increased expression of hypoxia-related genes and fibrin deposition in the liver from ALI patients with reduced ALT/LDH ratio. We also found intrahepatic hypoxia secondary to sinusoidal hypercoagulation in a mouse model of Concanavalin A (ConA)-induced ALI (18), where anticoagulation with recombinant human soluble thrombomodulin (rhTM) effectively reduced sinusoidal hypercoagulation and intrahepatic hypoxia, thereby leading to the improvement of liver injury. This study provides evidence that serum ALT/LDH ratio is clinically used as a biomarker of intrahepatic hypoxia in ALI patients and suggests that anticoagulation offers a novel therapeutic strategy for the treatment of ALF, which arises from sinusoidal hypercoagulation.

## Materials and methods

### 

#### Patients

This study was a retrospective single-center design. Patients with ALI, who had been admitted to Kyushu University Hospital between January 2005 and March 2018, were examined. ALI was defined as any syndrome that causes elevation of liver function tests for less than 6 months. Those who had serum ALT more than 200 U/l or serum total bilirubin (T. Bil.) more than 4 mg/dl or prothrombin time-international normalized ratio (PT-INR) over 1.2 at admission were enrolled, reaching up to 309. Blood tests for general hepatic function, coagulation ability, immunological variables such as IgG, IgA, IgM, ANA, smooth muscle antibodies, anti-mitochondrial antibody and liver-kidney microsomal antibodies (if required), and viral markers for hepatitis A virus (HAV), hepatitis B virus (HBV), hepatitis C virus (HCV), cytomegalovirus, herpes simplex virus (type 1 and 2) and EB virus were assessed using samples that had been obtained at admission. The diagnosis of patients with autoimmune hepatitis (AIH) was confirmed at their discharge according to the revised criteria of the International Autoimmune Hepatitis Group. For all patients diagnosed with AIH, pathological findings were required to fulfill the criteria. Patients with malignant tumors (n=20) and those with liver cirrhosis (n=10) were excluded. Ultrasound-guided percutaneous liver biopsies were performed in 17 patients with ALI. This study was approved by Kyushu University Hospital Ethics Committee (nos. 27-377 and 28-432). Informed consent of individual patients was not obtained because this study is of retrospective nature.

#### Animals

Eight-week-old male C57BL/6J mice weighing 20-25 g were obtained from Japan SLC (Shizuoka, Japan). Mice were maintained under controlled conditions with free access to standard chow and water. Mice were monitored via daily observations of health and behavior. All studies were performed in accordance with the Guide for the Care and Use of Laboratory Animals (National Institutes of Health) and approved by the Animal Care Committee of Kyushu University for three months starting in February 2020. Totally 186 mice were used in this study including preliminary experiments. All animals were euthanized by euthanasia under isoflurane at concentrations of 4-5% for induction and 2-3% for maintenance in accordance with the institutional guideline of the Animal Care Committee of Kyushu University. The depth of anesthesia was confirmed by loss of the postural reaction and righting reflex (the pedal withdrawal reflex in the forelimbs and hind limbs, the tail pinch reflex, and the eyelid reflex). Blood samples were drawn from tail vein or inferior vena cava and the livers were collected. Approximately 700-1,200 µl of blood was extracted by exsanguination. A combination of lack of pulse, breathing, corneal reflex, and presence of rigor mortis was used to confirm death. The blood samples were centrifuged for 15 min at 3,000 rpm (1,500 x g) at 4˚C, and serum samples were collected and stored at -80˚C. For RNA isolation and western blot analysis, liver samples were snap-frozen in liquid nitrogen and stored at -80˚C.

#### Experimental protocols

i) The ConA-induced ALI model mice (n=10). ConA (Sigma-Aldrich; Merck KGaA) was injected at 15 mg/kg via the tail vein (19). ii) The galactosamine (GalN)/tumor necrosis factor-α (TNF-α) (G/T)-induced ALI model mice (n=10). A total of 700 mg/kg of GalN (D-(+)-galactosamine hydrochloride; Sigma-Aldrich; Merck KGaA) was initially injected intraperitoneally. One hour after GalN injection, 15 µg/kg of TNFα (Recombinant human TNFα; Peprotech) was injected intravenously via the tail vein (20). iii) Control mice (n=5). The control mice were received saline via the tail vein. All animals were euthanized with isoflurane at 6 h after the injection of ConA or TNFα. Liver tissue samples were collected at 6 h after the injection of ConA or TNFα.

Anticoagulant-treated mice (n=4). rhTM was purchased from Asahi Kasei Pharma Co. Ltd. Upon ConA (15 mg/kg body weight) injection, rhTM (5 mg/kg body weight) dissolved in saline was injected intravenously via the tail vein (the ConA+TM group). The control animals received saline treatment at the time of ConA injection (the ConA group). The ConA group and ConA+TM group were sacrificed at 6 h (n=4, each group). To detect the hypoxic lesions in the liver, mice were injected intraperitoneally with pimonidazole (120 mg/kg; Chemicon) dissolved in saline 1 h before euthanasia.

#### Biochemical analysis

Normal ranges for human of ALT and LDH were 6-30 U/l and 119-229 U/l, respectively. In this study, we calculated ALT/LDH ratio with the following formula: ALT/LDH ratio=(serum ALT-ULN)/(serum LDH-ULN), where ULN stands for the upper limit of normal.

Blood samples of mice were withdrawn from the tail vein 6 h after the injection of ConA or TNFα. Serum levels of ALT, LDH, and fibrin degradation products (FDP) for mice were measured using chemical analyzer Fuji DRI-CHEM (Fuji Film) and FDP-ELISA kit (MyBioSource), respectively.

#### Immunohistochemical analyses (human)

Liver biopsy samples were fixed with 10% formalin and embedded in paraffin. Serial sections (5 µm) were cut from the blocks. Sinusoidal fibrin deposition was detected by phosphotungstic acid-hematoxylin (PTAH) staining. Paraffin-embedded liver sections were deparaffinized and rehydrated. Antigen retrieval was performed with Proteinase K (Dako) treatment. Endogenous peroxidase activity was blocked for 20 min with 3% hydrogen peroxide (Sigma-Aldrich; Merck KGaA). After blocking with diluted serum from the secondary antibodies host, the slides were incubated overnight (4˚C) with the following antibodies: Tissue factor (TF) antibodies (ab151748, Abcam, Cambridge, MA), hypoxia-inducible factor-1α (HIF-1α) antibody (NB100-105; Novus Biologicals), HIF-2α antibody (NB100-132; Novus Biologicals), lactate dehydrogenase-V (LDH-V) antibodies (ab9002; Abcam) and vascular endothelial growth factor A (VEGFA) antibodies (ab183100; Abcam). Secondary goat anti-rabbit or anti-mouse antibodies (Histofine Simple Stain kit; Nichirei Bioscience) was applied for 60 min at room temperature and stained with diaminobenzidine tetrahydrochloride (Nichirei Bioscience). The sections were counterstained with hematoxylin (Thermo Fisher Scientific, Inc.), dehydrated, and mounted. Positive areas in five randomly selected microscopic fields (magnification, x40) per section were measured using analysis software (BZ-X analyzer; Keyence) and the mean percentage of the positive area was calculated.

#### Histological examinations (Mice)

Liver tissue samples were collected at 6 h after the injection of TNF-α or ConA, fixed in 10% formalin and embedded in paraffin. Immunostaining was performed in the same way as human pathological examination. TF antibody (ab151748; Abcam), hypoxia-inducible factor (HIF)-1α antibodies (NB100-479; Novus Biologicals), HIF-2α antibodies (NB100-122; Novus Biologicals), LDH-V antibody (ab85472; Abcam) and vascular endothelial growth factor (VEGF)-A antibody (ab183100; Abcam) were used as the first antibody. Secondary goat anti-rabbit antibodies (Histofine Simple Stain kit; Nichirei Bioscience) was applied. The sections were visualized under a Keyence BZ-X700 microscope (Keyence). Positive areas in five randomly selected microscopic fields (x40 magnification) per section were measured using analysis software (BZ-X analyzer, Keyence, Osaka, Japan). For pimonidazole staining, Hypoxyprobe Omni kit (Abcam) was used according to the manufacturer's protocol (21). Positive areas in five randomly selected microscopic fields (magnification, x40) per section were measured using analysis software (BZ-X analyzer; Keyence) and the mean percentage of positive area was calculated.

#### Reverse transcription-quantitative PCR

Total RNA from liver tissue was isolated using TRIzol reagent (Invitrogen; Thermo Fisher Scientific, Inc.) and cDNA was synthesized from 500 µg RNA by GeneAmp RNA polymerase chain reaction (PCR) (Applied Biosystems). Quantitative polymerase chain reaction (qPCR) was performed using SYBR-Green on the ABI 7500 real-time PCR System (Applied Biosystems). The PCR reaction was carried out with a denaturation step at 95˚C for 30 sec, then 40 cycles at 95˚C for 5 sec and finally at 60˚C for 34 sec. To control for variations in the reactions, all data were normalized to GAPDH expression. Relative expression was presented using the 2^-^^Δ^^Ct^ method. The primer sequences are listed in Table I.

#### Western blot analysis

Aliquots of liver homogenate (30 µg) were separated by sodium dodecyl sulfate (SDS) polyacrylamide gels and transferred to polyvinylidene difluoride (PVDF) membranes. Nonspecific binding was blocked with 5% nonfat milk for one hour and incubated overnight at 4˚C with primary antibodies: HIF-1α antibodies (NB100-479; Novus Biologicals), HIF-2α antibodies (NB100-102; Novus Biologicals) and anti-β-actin antibodies (ab16039; Abcam). Membranes were washed with PBS with Tween-20 (PBST) three times for 10 min and then incubated with a secondary goat anti-rabbit antibodies (1:5,000) for one hour at 37˚C. Finally, the membranes were washed with PBST three times for 10 min and developed with the ECL system (GE Healthcare).

#### Statistical analysis

Data were analyzed using JMP Pro Version 11 statistical software (SAS Institute Inc.). The results were expressed as the means and standard deviation (SD) or standard error of the means (SEM), or Median and inter-Quartile range. Significant differences between groups were assessed using the χ^2^-square test and unpaired Student's t-test. The differences of means among multiple groups were analyzed by using one-way ANOVA and Tukey's post hoc test. P<0.05 was considered to indicate a statistically significant difference.

## Results

### 

#### Characteristics of ALI patients classified by serum ALT/LDH ratio

**Figure 1:**
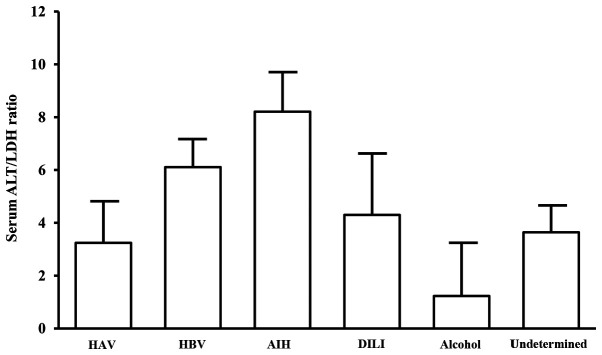
Serum ALT/LDH4 ratio in every etiology. ALT/LDH, aminotransferase/lactate dehydrogenase ratio.

An ALT/LDH ratio of 1.5 was used to diagnose hypoxic hepatitis, which is characterized by intrahepatic hypoxia and massive liver damage as a result of cardiac failure-induced reduction of oxygen delivery (22). In this study, when coagulopathy was defined as FDP >10 µ/ml, ROC analysis showed the cut-off value of serum ALT/LDH to be 1.48 (Fig. S1). We, therefore, used 1.5 as the cut-off value to identify ALI patients with sinusoidal hypercoagulation. It is likely that patients with ALT/LDH ratio ≤1.5 (i.e., the low ALT/LDH ratio group) were complicated with microcirculatory disturbance relative to those with ALT/LDH ratio >1.5 (i.e., the high ALT/LDH ratio group). Accordingly, we classified our ALI patients into the low ALT/LDH ratio and high ALT/LDH ratio groups, based upon ALT/LDH ratio of 1.5 (Table II). There were substantial numbers of HBV and AIH patients in the high ALT/LDH ratio group (12 HAV, 55 HBV, 35 AIH, 9 drugs, 5 alcoholic, 36 undetermined etiologies, and 12 others). On the other hand, HAV patients and those with undetermined etiologies were mostly in the low ALT/LDH ratio group (22 HAV, 16 HBV, 4 AIH, 6 drugs, 13 alcoholic, 40 undetermined etiologies, and 14 others). The AIH patients showed the highest ALT/LDH ratio, whereas HAV patients exhibited lower ALT/LDH ratio (Fig. 1). Moreover, ALT (P<0.0001), LDH (P<0.0001), ferritin (P<0.0001), and MELD (P=0.0002) score were significantly higher in the low ALT/LDH ratio group than those in the high ALT/LDH ratio group, while platelet count (P=0.0096) and T. Bil. (P=0.0037) were significantly higher in the high ALT/LDH ratio group. Importantly, the low ALT/LDH ratio group showed significantly increased levels of PT-INR (P=0.0191) and FDP (P<0.0001) relative to the high ALT/LDH ratio group (Table II).

#### Sinusoidal hypercoagulation and intrahepatic hypoxia in liver biopsy samples

**Figure 2:**
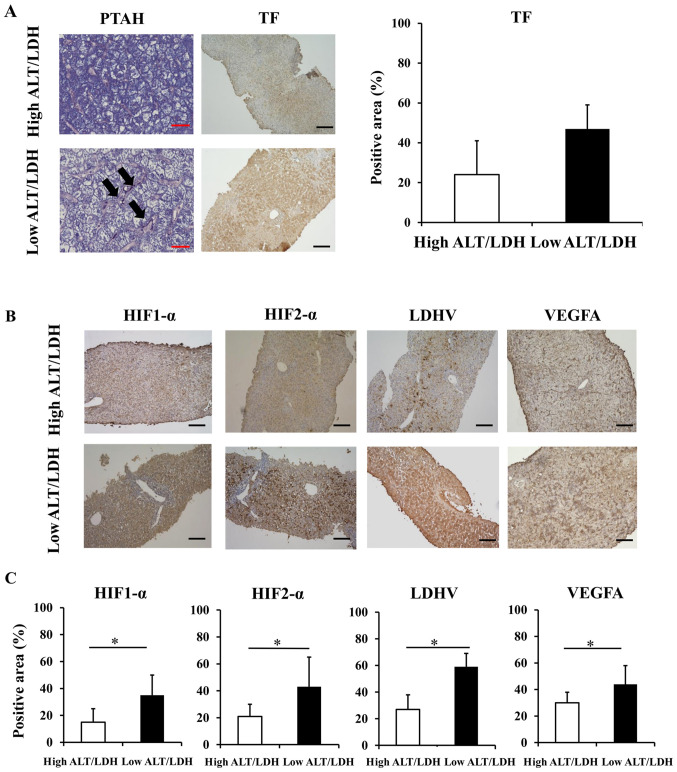
Histological evaluation of intrahepatic hyper-coagulation and hypoxia related proteins of 17 patients with ALI. (A) TF staining (magnification, x100) and phosphotungstic acid-hematoxylin staining (magnification, x400) of liver sections in a patient with low ALT/LDH ratio and a patient with high ALT/LDH ratio were performed to evaluate sinusoidal coagulopathy. The arrows indicate fibrin depositions in sinusoids. Black Scale bar=100 µm. Red Scale bar=25 µm. (B) HIF-1α, HIF-2α, LDH-V and VEGFA staining of liver sections in a patient with low ALT/LDH ratio and a patient with high ALT/LDH ratio were performed to evaluate intrahepatic hypoxia (magnification, x100). Black Scale bar=100 µm. (C) The percentage of positive area in HIF-1α, HIF-2α, LDH-V and VEGFA staining of liver sections. ^*^P<0.05. ALI, acute liver injury; ALT/LDH, aminotransferase/lactate dehydrogenase ratio; HIF, hypoxia-inducible factor; LDH-V, lactate dehydrogenase-V; TF, tissue factor.

Liver biopsy samples were obtained from 17 patients (2 HAV, 6 AIH, 2 drugs, 7 undetermined etiologies) (Table III). Fibrin deposition, a hallmark of sinusoidal hypercoagulation (10,23), was diffusely distributed in the low ALT/LDH ratio group but barely detected in the high ALT/LDH ratio group (Fig. 2A). We also found increased protein expression of TF, a cell surface glycoprotein that plays a role in the initiation of sinusoidal coagulopathy (24), in the low ALT/LDH ratio group relative to high ALT/LDH ratio group; the immune-positive area was significantly higher in the low ALT/LDH ratio group (47±13%) than in the high ALT/LDH ratio group (25±17%) (P=0.01). We also performed immunohistochemical analysis of hypoxia-related proteins. Both HIF-1α and HIF-2α, key transcriptional factors for hypoxic response, were strongly positive in the low ALT/LDH ratio group relative to the high ALT/LDH ratio group, with immune-positive area being significantly extended (Fig. 2B and C). There was increased protein expression of LDH-V and VEGFA in the low ALT/LDH ratio group relative to high ALT/LDH ratio group (Fig. 2B and C). Increased expression of hypoxia-related proteins in the liver from the low ALT/LDH ratio group is consistent with the notion that intrahepatic hypoxia develops in the liver with low ALT/LDH ratio group, which suggests that serum ALT/LDH ratio is useful to evaluate intrahepatic hypoxia in the liver.

#### Sinusoidal hypercoagulation and intrahepatic hypoxia in mouse models of ALI

**Figure 3:**
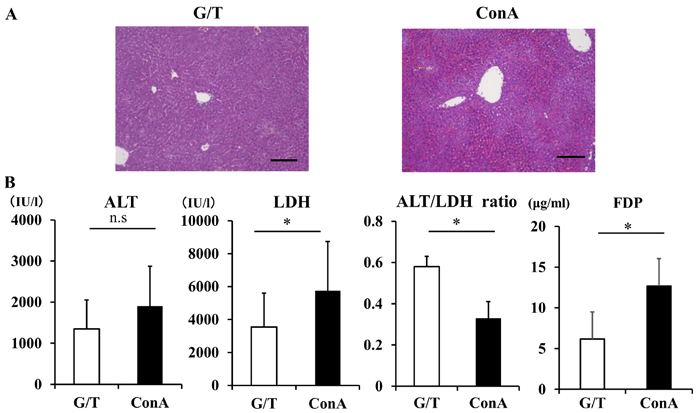
Serum ALT/LDH ratio and FDP in ALI model mice. (A) Hematoxylin and eosin staining of liver sections. Scale bar=100 µm. (B) Serum ALT, LDH, ALT/LDH ratio and FDP levels. Data are expressed as the mean ± SD. ^*^P<0.05, ns, non-significant. ALT/LDH, aminotransferase/lactate dehydrogenase ratio; FDP, fibrin degradation products; ALI, acute liver injury; ConA, Concanavalin A.

We next examined sinusoidal hypercoagulation and intrahepatic hypoxia in 2 different murine models of human ALI. The ConA-induced ALI is a T-cell-driven liver injury model, where cytotoxic effector molecules are thought to play a key role in the development of cell death (25,26), whereas the G/T-induced ALI represents an apoptotic model of liver injury, thus resembling human acute viral hepatitis (27). Histological analysis of liver sections revealed inflammation and piecemeal necrosis with robust sinusoidal congestion in ConA mice and spotty hemorrhagic legions in G/T mice (Fig. 3A). In this study, peak value of serum ALT in G/T-treated mice were slightly lower than those in ConA-treated mice, with no statistically significant difference (Fig. 3B). On the other hand, serum LDH was significantly elevated in ConA-treated mice relative to G/T-treated mice (G/T mice vs. ConA mice; 3541.2±2069.1 vs. 5740.0±3005.1 IU/l, P=0.04). Consequently, serum ALT/LDH ratio was significantly lower in ConA-treated mice than G/T-treated mice (G/T mice vs. ConA mice; 0.48±0.05 vs. 0.33±0.08, P=0.04). Serum FDP in ConA-treated mice was significantly higher than those in G/T-treated mice (G/T mice vs. ConA mice: 6.19±3.33 vs. 12.67±3.38 µg/ml, P=0.01) (Fig. 3B).

**Figure 4:**
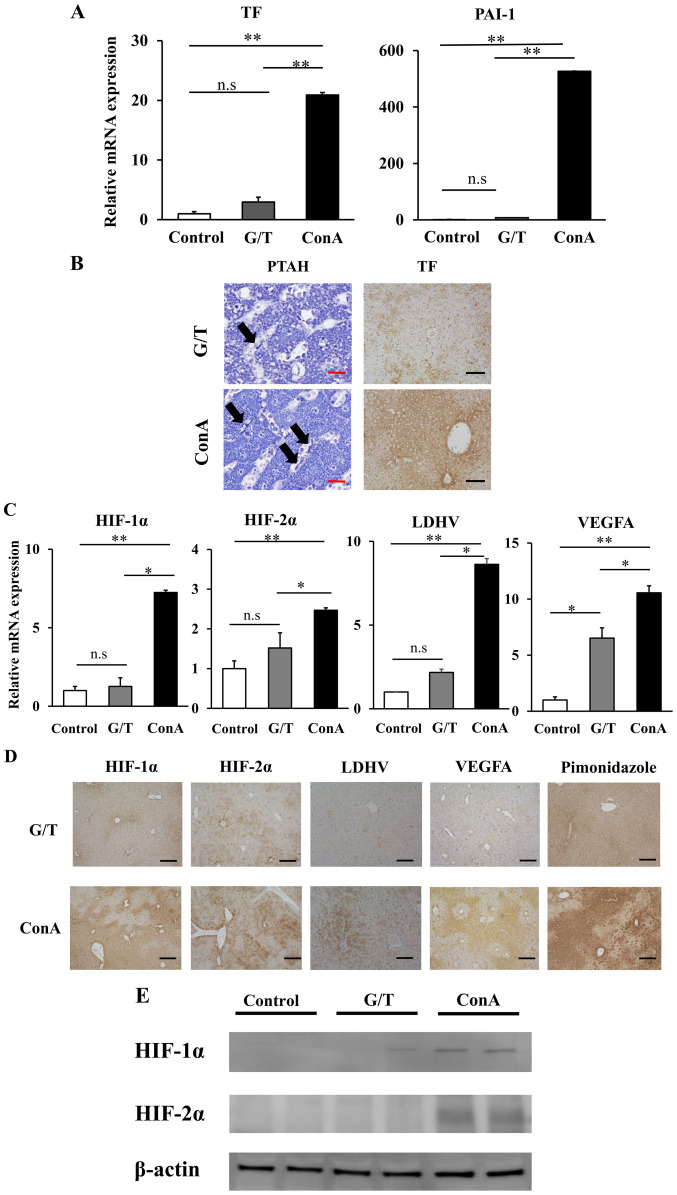
Intrahepatic hyper-coagulation and hypoxia related proteins expressions of ALI model mice. (A) Hepatic mRNA expression of TF and PAI-1 were quantified by RT-q PCR. Data are expressed as the mean ± SE (control mice, n=5; G/T induced ALI model mice, n=5; ConA induced ALI model mice, n=5). (B) TF staining (magnification, x100) and PTAH staining (magnification, x400) of GT and ConA induced ALI model mice liver. The arrows indicate fibrin depositions in sinusoids. Black Scale bar=100 µm. Red Scale bar=5 µm. (C) Hepatic mRNA expression of HIF-1α, HIF-2α, LDHA and VEGFA were quantified by RT-qPCR. Data are expressed as the mean ± SE (control mice, n=5; G/T induced ALI model mice, n=5; ConA induced ALI model mice, n=5). (D) HIF-1α, HIF-2α, LDH-V, VEGFA and pimonidazole staining of GT and ConA induced ALI model mice liver were performed to evaluate intrahepatic hypoxia (magnification, x100). Black Scale bar=100 µm. (E) Western blot analysis showing the levels of expression of HIF-1α and HIF-2α in GT and ConA induced ALI model mice liver. ^*^P<0.05, ^**^P<0.01. ns, non-significant. ALI, acute liver injury, RT-q, reverse-transcription quantitative; PAI-1, plasminogen activation inhibitor-1; ConA, Concanavalin A; PTAH, phosphotungstic acid-hematoxylin; HIF, hypoxia-inducible factor; LDH, lactate dehydrogenase; TF, tissue factor.

Hepatic mRNA expression of TF and plasminogen activation inhibitor-1 (PAI-1) were markedly higher in ConA mice than those in G/T mice (Fig. 4A). Histological examination showed strong lobular expression of TF and diffuse distribution of fibrin in ConA mice, but scarcely detected in G/T mice (Fig. 4B). Hepatic mRNA expression of HIF-1/2α, LDH-V, and VEGFA was also significantly upregulated in ConA mice relative to G/T mice (Fig. 4C). Immunohistochemical analysis revealed their strong expression in the liver from ConA-treated mice (Fig. 4D, E). The hypoxic area was diffusely distributed in ConA mice, but faintly detected in G/T mice as revealed by pimonidazole immunostaining (Fig. 4D). These observations, taken together, suggest the development of intrahepatic hypoxia and sinusoidal hypercoagulation in ConA mice relative to G/T mice.

#### Therapeutic effect of anticoagulant in the ConA-induced mouse model of ALI

**Figure 5:**
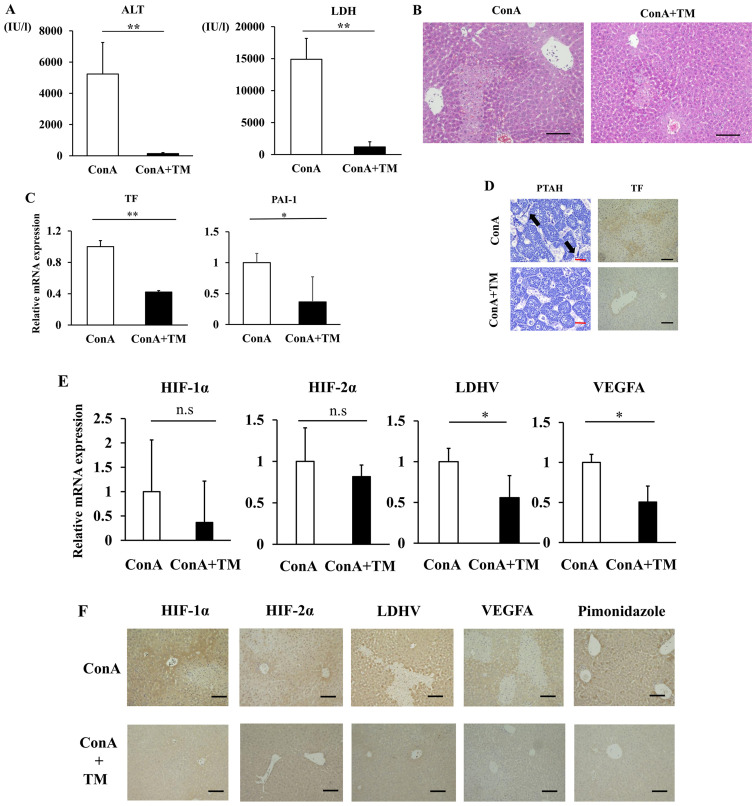
rhTM suppresses liver damage in ConA-induced ALF model mice. (A) Serum ALT and LDH. Data are expressed as the mean ± SD. (B) Hematoxylin and eosin staining of liver sections (magnification, x100). Scale bar=100 µm. (C) Hepatic mRNA expression levels of TF and PAI-1 were quantified by RT-qPCR. Data are expressed as the mean ± SE. (D) TF staining (magnification, x100) and phosphotungstic acid-hematoxylin staining (magnification, x400) of liver. The arrows indicate fibrin depositions in sinusoids. Black Scale bar=100 µm. Red Scale bar=5 µm. (E) Hepatic mRNA expression levels of HIF-1α, HIF2α, LDHA and VEGFA were quantified by RT-qPCR. Data are expressed as the mean ± SE. (F) HIF-1α, HIF-2α, LDH-V, VEGFA and pimonidazole staining of liver were performed to evaluate intrahepatic hypoxia (magnification, x100). Black Scale bar=100 µm. ^*^P<0.05, ^**^P<0.01. ns, non-significant. rhTM, human soluble thrombomodulin; ConA, Concanavalin A; ALF, Acute liver failure; ALT, aminotransferase; LDH, lactate dehydrogenase ratio; PAI-1, plasminogen activation inhibitor-1; RT-q, reverse-transcription quantitative; HIF, hypoxia-inducible factor; TF, tissue factor.

Given that ConA mice develop sinusoidal hypercoagulation and intrahepatic hypoxia during the progression of ALF, we examined the therapeutic effect of anticoagulant rhTM in the ConA-induced mouse model of ALI. Surprisingly, the ConA+TM group showed significantly decreased serum ALT and LDH relative to the vehicle-treated ConA group (Fig. 5A). Histologically, extensive necrosis was observed in the ConA group, while necrotic area was obviously smaller in the ConA+TM group (Fig. 5B). Hepatic mRNA expressions of TF and PAI-1 were downregulated in the ConA+TM group relative to the ConA group (Fig. 5C). TF staining was lower in the ConA+TM group than that in the ConA group, and diffuse sinusoidal fibrin deposition observed in the ConA group was abolished by rhTM administration (the ConA+TM group) (Fig. 5D). In addition, rhTM treatment significantly reduced hepatic expression of LDHV and VEGFA (Fig. 5E and F). Immunostaining positive area of HIF1-α and HIF2-α were lower in the ConA+TM group than that in the ConA group (Fig. 5F). Hepatic mRNA expression of HIF1-α and HIF2-α in the ConA+TM group tended to be reduced relative to the ConA group, with no statistical significance. Importantly, anticoagulant treatment with rhTM resulted in the reduction of hypoxic area detected by pimonidazole staining relative to the ConA group (Fig. 5F).

## Discussion

Sinusoidal microcirculatory disturbance has been thought to be involved in the pathogenesis of ALF (5). However, it is currently unclear whether it modulates the onset and progression of liver diseases and if so, how it occurs in any etiologies. In this study, we classified ALI patients into two groups by serum ALT/LDH ratio; the low ALT/LDH ratio and high ALT/LDH ratio groups, and found upregulated FDP and PT-INR and sinusoidal fibrin deposition in the low ALT/LDH ratio group relative to the high ALT/LDH ratio group. Because these findings were also accompanied with enhanced hepatic expression of hypoxia-related genes, it is likely that intrahepatic hypoxia develops as a result of sinusoidal microcirculatory disturbance in the livers in the low ALT/LDH ratio group. Complication with systemic coagulopathies such as disseminated intravascular coagulation could also increase FDP and PT-INR, however, fibrin deposition observed locally in the liver suggests that sinusoidal hypercoagulation is responsible for the apparent increase in systemic coagulation. Because LDH is known to be induced under the hypoxic condition, serum ALT/LDH ratio can be a noninvasive surrogate to evaluate the involvement of intrahepatic hypoxia in ALI. However, there is no direct evidence for intrahepatic hypoxia in humans. Cassidy and Reynolds previously showed that the cut-off value of 1.5 for ALT/LDH ratio can be used to diagnose the hypoxic hepatitis, which is characterized by massive liver damage in patients with severe heart failure (22). In this study, using the cut-off value of 1.5 for serum ALT/LDH ratio, we successfully classified ALI patients with and without sinusoidal hypercoagulation.

To further investigate the correlation between sinusoidal hypercoagulation and intrahepatic hypoxia, we analyzed two different animal models of ALI: ConA mice and G/T mice. Concanavalin A is known to stimulate T-lymphocyte mediated immune activation and thus promote massive liver injury enhanced by sinusoidal hypercoagulation (18,25,28). G/T mice represents parenchymal cell apoptosis model of liver injury (27). Many therapeutic approaches have been tested in these models; however, little was examined for sinusoidal hypercoagulation and intrahepatic hypoxia. In this study, we found increased expressions of TF and PAI-1 and histological fibrin deposition in ConA mice but barely observed in G/T mice. The pimonidazole-stained hypoxic area as well as upregulation of hypoxia-related genes were markedly extended in ConA mice relative to G/T mice. Moreover, histological findings in ConA mice are similar to ALI patients in the low ALT/LDH ratio group. These observations, taken together, suggest that liver damages in the low ALT/LDH ratio group as well as ConA mice are largely enhanced by intrahepatic hypoxia as a result of sinusoidal hypercoagulation.

In this study, we demonstrated that ALI patients in the low ALT/LDH ratio show sinusoidal hypercoagulation and intrahepatic hypoxia. MELD score was higher in the low ALT/LDH ratio groups than the high ALT/LDH ratio group, but there was no significant difference in prognosis between the high and low ALT/LDH ratio groups (P=0.47). Interestingly, HAV and undetermined etiologies were mostly classified in the low ALT/LDH ratio group, while AIH and HBV were in the high ALT/LDH ratio group. We analyzed the history of the patients with undetermined etiologies, but there was nothing of note. While ALI patients with HAV barely progress to liver failure, those with HBV and AIH show a poor prognosis and occasionally require liver transplantation (29-31). Hepatocyte cell death occurs in the livers from ALI, and there is evidence that skewing cell death toward apoptosis is correlated to poor outcome (32-35). Because apoptosis is an energy-consuming process, hypoxia impairs ATP production and thus shifts cell death toward necrosis (36). It is conceivable that sinusoidal hypercoagulation and intrahepatic hypoxia in the low ALT/LDH ratio group might induce necrosis-dominant cell death, which is correlated with a favorable prognosis of the low ALT/LDH ratio group (HAV) relative to high ALT/LDH ratio group (AIH and HBV). Kato *et al* previously reported that proinflammatory signals elicited by IFN-γ and TNFα in both hepatic macrophages and sinusoidal endothelial cells are important for the development of sinusoidal hyper coagulation in ConA mice (18). The immune reactions including specific set of the etiology-dependent proinflammatory cytokines might provide particular pathology and prognosis of ALI (37-40).

In this study, anticoagulation therapy using rhTM reduced liver damages in ConA mice, which is consistent with our previous report of the beneficial effect of rhTM on acetaminophen-induced ALI mice (41). Given that acetaminophen-induced ALI is known to trigger sinusoidal hypercoagulation, the data of this study suggest that sinusoidal hypercoagulation is responsible for the impaired hepatic microcirculation, and anticoagulation therapy can attenuate liver damage probably by blood reperfusion. Therefore, anticoagulation therapy might be useful in ALI patients with intrahepatic hypoxia as a result of sinusoidal hypercoagulation, who are classified into the low ALT/LDH ratio group.

The limitation of this study is the small number of liver biopsy samples and wide spectrum of background for ALI patients. For that reason it is not completely certified that serum ALT/LDH ratio reflect sinusoidal hyper coagulation and intrahepatic hypoxia, however correlation of serum marker and histological examination for human and mouse samples might complement the finding.

In conclusion, we demonstrate that serum ALT/LDH ratio helps to identify ALI patients with intrahepatic hypoxia as a result of sinusoidal hypercoagulation. Our data also provide evidence that sinusoidal hypercoagulation precedes intrahepatic hypoxia during the course of ALI and thus offer a novel therapeutic strategy, which might produce appropriate treatment selection and better prognostic implication.

## Supplementary Material

ROC Curves for Coagulopathy Defined as FDP >10 mg/ml based upon serum ALT/LDH Ratio. ROC was performed for coagulopathy defined as FDP >10 mg/ml based on ALT/LDH ratio. Area under the curve (AUC) was 0.77. ROC, Receiver Operating Characteristic; FDP, fibrin degradation products; ALT, aminotransferase; LDH, lactate dehydrogenase ratio.

## Figures and Tables

**Table I:** Reverse-transcription quantitative PCR primer sequences.

	Primer sequences	
Gene	Forward (5'-3')	Reverse (5'-3')
GAPDH	TACCCCCAATGTGTCCGTC	GGTCCTCAGTGTAGCCCAAG
TF	TGCTTCTCGACCACAGACAC	TAAAAACTTTGGGGCGTTTG
PAI-1	TCTGGGAAAGGGTTCACTTTACC	GACACGCCATAGGGAGAGAAG
HIF1-α	GTATTATTCAGCACGACTT	GACATTGCCAGGTTTAT
HIF2-α	CTGAGGAAGGAGAAATCCCGT	TGTGTCCGAAGGAAGCTGATG
LDHV	TGGCGACTCCAGTGTGCCTG	AGGCACTGTCCACCACCTGCT
VEGFA	CTGTGCAGGCTGCTGTAACG	GTTCCCGAAACCCTGAGGAG

TF, Tissue factor; PAI-1, plasminogen activator inhibitor-1; HIF1-α, hypoxia-inducible factor 1-α; HIF2-α, hypoxia-inducible factor 2-α; LDHV, lactate dehydrogenase V; VEGFA, vascular endothelial growth factor A.

**Table II:** Characteristics of patients with ALI upon admission, classified according to ALT/LDH ratio.

Characteristic	Total	High ALT/LDH ratio (ALT/LDH>1.5)	Low ALT/LDH ratio (ALT/LDH≤1.5)	P-value
N	279	164	115	
Age	45 (34-58)	45 (34-60)	45 (35-55)	0.5890
Sex (M/F)				0.7180
Male	154	92	62	
Female	125	72	53	
Etiology (%)				<0.0001
HAV	34 (12.2)	12 (7.3)	22 (19.1)	
HBV	71 (25.4)	55 (33.5)	16 (13.9)	
AIH	39 (13.9)	35 (21.3)	4 (3.5)	
DILI	15 (5.3)	9 (5.4)	6 (5.2)	
Alcohol	18 (6.4)	5 (3.1)	13 (11.3)	
Undetermined	76 (27.2)	30 (18.3)	40 (34.8)	
Others	24 (8.6)	15 (9.1)	9 (7.8)	
Platelet (x10^3^/µl)	14.2 (9.6-19.1)	15.6 (10.7-19.98)	12.4 (8.6-16.6)	0.0096
FDP (µg/ml)	10.8 (4.4-24.4)	6.25 (2.6-11.58)	19.3 (11.4-44)	<0.0001
PT-INR	1.78 (1.41-2.37)	1.57 (1.28-2.17)	1.97 (1.63-2.76)	0.0191
Alb (g/dl)	3.5 (3.1-3.9)	3.5 (3.1-3.9)	3.5 (3.1-3.9)	0.9353
Cre (mg/dl)	0.73 (0.56-1.02)	0.675 (0.54-0.82)	0.84 (0.61-1.61)	0.0023
TB (mg/dl)	4.8 (2.6-11.1)	6.2 (3.33-12.78)	3.8 (2-7.3)	0.0037
AST (IU/l)	1848 (517-5227)	1216.5 (515.75-2696.5)	4793 (517-9507)	<0.0001
ALT (IU/l)	2349 (680-4365)	1670 (725.5-3338)	3232 (324-6109)	<0.0001
LDH (IU/l)	783 (386-3310)	526 (358.75-977.25)	3669 (879-8350)	<0.0001
Ferritin (ng/ml)	3890.6 (914.7-16955)	2676.1 (562.75-7130.5)	11980.5 (1846.1-49762)	<0.0001
NH3 (µg/dl)	63 (49-96)	61 (49-86.5)	66 (47.5-105)	0.1734
MELD	16.78 (11.33-23.71)	15.52 (10.15-20)	18.79 (13-28)	0.0002
Survive/death and LT	224/55	134/30	90/25	0.4700

Data are expressed as median and interquartile range. ALI, acute liver injury; HAV, hepatitis A virus; HBV, hepatitis B virus; AIH, autoimmune hepatitis; DILI, drug-induced liver injury; LT, liver transplantation; FDP, fibrin/fibrinogen degradation products; PT-INR, prothrombin time international normalized ratio; Alb, albumin; Cre, creatinine; TB, total bilirubin; AST, aspartate aminotransferase; ALT, alanine aminotransferase; LDH, lactate dehydrogenase; NH3, ammonia; MELD, model for end-stage liver disease.

**Table III:** Etiology and laboratory findings for 17 patients with ALI in which liver biopsy was performed.

		Blood chemistry					Positive area (%)				
Case	Etiology	ALT (IU/l)	LDH (IU/l)	ALT/LDH ratio	PT-INR	FDP (µg/ml)	TF	HIF1-α	HIF2-α	LDHV	VEGFA
1	Undetermined	7,711	9,172	0.86	1.3	39.8	45	39	38	55	60
2	Undetermined	4,315	8,800	0.50	1.67	10	60	57	70	72	62
3	HAV	4,409	4,501	1.03	1.24	24.4	51	48	63	52	44
4	Undetermined	4,495	4,105	1.15	1.76	52.3	60	30	17	62	24
5	Undetermined	4,973	3,201	1.66	1.41	12	40	26	30	42	40
6	Undetermined	5,769	2,257	2.83	2.32	25.5	60	28	23	43	25
7	HAV	1,020	1,253	0.97	1.42	48.9	42	25	56	70	40
8	Undetermined	5,344	825	8.92	2.05	16	35	38	35	40	32
9	AIH	2,780	632	6.82	1.31	5.9	5	10	13	25	41
10	AIH	1,198	573	3.40	2.24	5.1	4	8	15	24	33
11	DILI	1,220	560	3.60	1.25	15.4	26	10	13	27	20
12	AIH	337	452	1.38	1.51	2.5	26	15	19	47	34
13	DILI	561	364	3.93	1.22	2.5	12	5	10	13	14
14	AIH	389	290	5.89	1.22	8	16	12	12	15	28
15	Undetermined	2,621	281	49.83	1.49	2.5	38	17	32	36	36
16	AIH	680	197	20.31	1.26	2.5	26	8	30	23	29
17	AIH	680	190	16.67	1.48	2.5	10	8	22	16	33

TF, tissue factor; HIF1-α, hypoxia-inducible factor 1-α; HIF2-α, hypoxia-inducible factor 2-α; LDHV, lactate dehydrogenase V; HAV, hepatitis A virus; AIH, autoimmune hepatitis; DILI, drug-induced liver injury; ALT, alanine aminotransferase; LDH, lactate dehydrogenase; PT-INR, prothrombin time international normalized ratio; FDP, fibrin/fibrinogen degradation products.

## Data Availability

The dataset used and/or analyzed during the current study are available from the corresponding author on reasonable request.
