# Supplementary material for: Microcirculatory disturbance in acute liver injury
Source: Exp Ther Med. 2021 Apr 9;21(6):596. doi: 10.3892/etm.2021.10028 (PMC8056117; doi:10.3892/etm.2021.10028)

Figure S1. ROC Curves for Coagulopathy Defined as FDP >10 mg/ml based upon serum ALT/LDH Ratio. ROC was performed for coagulopathy defined as FDP >10 mg/ml based on ALT/LDH ratio. Area under the curve (AUC) was 0.77. ROC, Receiver Operating Characteristic; FDP, fibrin degradation products; ALT, aminotransferase; LDH, lactate dehydrogenase ratio.

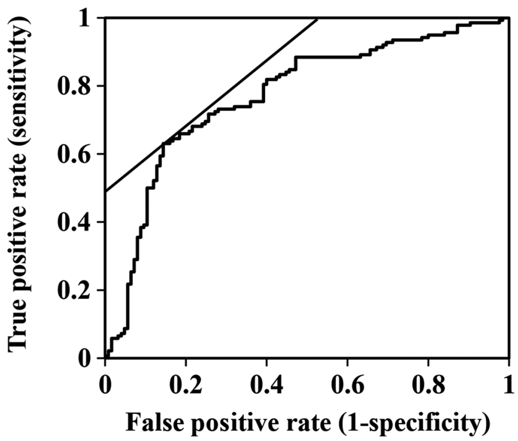

Supplement: ROC Curves for Coagulopathy Defined as FDP >10 mg/ml based upon serum ALT/LDH Ratio. ROC was performed for coagulopathy defined as FDP >10 mg/ml based on ALT/LDH ratio. Area under the curve (AUC) was 0.77. ROC, Receiver Operating Characteristic; FDP, fibrin degradation products; ALT, aminotransferas [file Supplementary_Data.pdf]
